# Long-Term Health-Related Quality of Life in People Living with HIV Who Present to Care with AIDS or Severe Immunodeficiency: The CoRIS AIDS Survivors Study

**DOI:** 10.1007/s10461-025-04730-x

**Published:** 2025-05-15

**Authors:** Julia Portilla-Tamarit, María José Fuster-RuizdeApodaca, Irene Portilla-Tamarit, Sergio Reus, Melchor Riera, Nuria Espinosa, Juan Martín, Enrique Bernal, Mar Masia, Sonia Calzado, Joaquín Portilla

**Affiliations:** 1 Infectious Diseases Unit and Internal Medicine Department, University General Hospital Dr, Balmis, Alicante, Spain; 2 Alicante Institute for Health and Biomedical Research (ISABIAL), Alicante, Spain; 3 Spanish AIDS Research Network, Carlos III Health Institute, Madrid, Spain; 4 Clinical Medicine Department, Miguel Hernandez University, Elche, Alicante, Spain; 5 Faculty of Psychology, UNED, Madrid, Spain; 6 Spanish Interdisciplinary AIDS Society (SEISIDA), Madrid, Spain; 7 Department of Health Psychology, University of Alicante, San Vicente del Raspeig Street CP 03690 San Vicente, Alicante, Spain; 8 Son Espases University Hospital, Palma de Mallorca, Spain; 9 Clinical Unit of Infectious Diseases, Clinical Microbiology and Parasitology, Institute of Biomedicine of Seville/Virgen del Rocio University Hospital, CSIC/University of Seville, Seville , Spain; 10 12th of October University Hospital, Madrid, Spain; 11 Reina Sofía Hospital, Murcia, Spain; 12 University General Hospital of Elche, Elche, Spain; 13 University Hospital Parc Taulí, Sabadell, Spain

**Keywords:** Health-related quality of life, HIV/AIDS, Advanced HIV disease, Survival, CD4 cells, Immunodeficiency

## Abstract

Our aim was to evaluate long-term health-related quality of life (HRQoL) in 253 people living with HIV (PLH) from the CoRIS cohort presenting to care with advanced HIV disease (AIDS or CD4 ≤ 100 cells/µL) and who had survived ≥ 5 years. Participants completed the WHOQOL-HIV-BREF and EQ-5D-5 L questionnaires. Clinical and immunological data were provided by the CoRIS at enrollment and on questionnaire completion. Linear repeated measures analyses assessed the evolution of immunological markers. Partial least squares structural equation modeling showed the longitudinal impact of baseline immunological markers on HRQoL. High baseline CD4 counts predicted higher WHOQOL-HIV-BREF independence scores (*p* = 0.021) and a marginally higher EQ-5D-5 L index value (*p* = 0.058), which was also associated with CD8 (*p* = 0.015). A higher CD4/CD8 ratio predicted lower scores on the WHOQOL-HIV-BREF ‘spirituality, religion and personal belief’ dimension (*p* = 0.006). Currently, PLH who present with advanced HIV disease achieve a moderate long-term HRQoL, with room for improvement.

**Supplementary Information:**

The online version contains supplementary material available at 10.1007/s10461-025-04730-x.

## Resumen

Evaluamos la calidad de vida relacionada con la salud (CVRS) en personas con VIH (PVIH) que han sobrevivido a un diagnóstico de enfermedad avanzada por VIH, identificando factores asociados con la CVRS a largo plazo. Incluimos 253 participantes con diagnóstico de sida y/o CD4 + ≤ 100/µL al momento de su inclusión y que permanecieron vivos en la cohorte durante ≥ 5 años. Los participantes completaron los cuestionarios WHOQOL-HIV-BREF y EQ-5D-5 L, obteniendo datos clínicos e inmunológicos al momento de la inclusión (t0) y durante la realización de los cuestionarios de CVRS (t1). Realizamos análisis de medidas lineales repetidas para evaluar la evolución de los marcadores inmunológicos. Utilizamos el modelo de ecuaciones estructurales parcial por mínimos cuadrados (PLS-SEM) para predecir el impacto longitudinal de los marcadores inmunológicos en el momento inicial (t0) sobre la CVRS (t1). Un elevado recuento de CD4 + al diagnóstico predijo una puntación más elevada en la dimensión “nivel de independencia” (WHOQOL-HIV-BREF) (*p* = 0,021) y una puntuación marginalmente más alta (*p* = 0,058) en el índice de valor del EQ-5D-5 L, también relacionada con los niveles de CD8 (*p* = 0,015). El cociente CD4/CD8 fue predictivo de puntuaciones más bajas en la dimensión de espiritualidad/creencias personales (*p* = 0,006). En la actualidad, las PVIH que presentan enfermedad avanzada al diagnóstico logran una calidad de vida normal, con margen de mejora.

## Introduction

Before the advent of antiretroviral therapy (ART), people living with HIV (PLH) who presented to care with an AIDS diagnosis or severe immunodeficiency had a poor prognosis and a high risk of early mortality [1]. During the early years of the AIDS epidemic, people living with the disease endured loneliness, social isolation, stigma, depression, and other mental and physical health disorders [2]. PLH who survive an AIDS diagnosis are currently known as long-term HIV survivors or AIDS survivors [1, 3, 4].

More than 40 years after the first reported cases, AIDS remains a global health problem [5, 6, 7]. In Spain, despite free access to HIV tests and clinical care, there were 311 cases of AIDS reported in 2022, which represents an approximate incidence of 0.80 per 100,000 inhabitants. The median age at AIDS diagnosis was 45 years (interquartile range [IQR] 36–52 years). Most of these new cases were in men (81.9%), 33.8% were in men who have sex with men, 33.2% occurred in heterosexual men and women, and 4.8% in intravenous drug users [8]. The overall mortality rate from HIV and AIDS was 0.6 per 100,000 inhabitants [9].

The model of HIV care has undergone a profound transformation since the 1980s. Initial goals included reducing mortality, improving immune status, and providing end-of-life care. Subsequently, the focus of care evolved to reducing treatment toxicities, maintaining viral suppression, and improving life expectancy. Today, the aim of treatment is to achieve a good quality of life, centered on patient values and covering the broad spectrum of patient profiles [10]. Health-related quality of life (HRQoL) is an essential component of long-term health. It is a multidimensional concept that encompasses the impact of health on various areas of well-being [11]. Longitudinal studies have shown that health markers impact quality of life, and that quality of life also has a long-term impact on health. Indeed, HRQoL appears to be a predictor of morbidity, hospitalizations, and mortality [12, 13]. HRQoL determinants include virological and immunological markers, comorbidities, and advanced HIV disease [14, 15].

The World Health Organization (WHO) defines advanced HIV disease (AHD) as WHO clinical Stage 3 or 4 disease and low CD4 cells/µL (< 200, < 100 or < 50) in adults [16]. Those with a CD4 count below 100 cells/µL have been defined as severely immunosuppressed and require special care [17, 18]. Compared with PLH who are diagnosed early, those presenting with AHD are at greater risk of AIDS events, non-AIDS events (NAEs), reduced life expectancy, and increased mortality [19, 20, 21, 22, 23]. Moreover, PLH with AHD have poorer HRQoL than PLH without AIDS or with higher CD4 counts [12, 24, 25, 26], particularly in the dimensions of physical and mental health [14, 27, 28]. The people most vulnerable to developing and presenting with AHD are intravenous drug users, migrants, older people, and people with low socioeconomic status, lower educational levels, and social or internalized stigma [29, 30, 31, 32, 33]. All these conditions have also been associated with lower HRQoL [12, 15, 34]. People with AHD require special medical care, social support, and interventions targeted at improving their HRQoL [17, 18, 19, 35]. While there are many HRQoL studies in cancer survivors, we have found no data on the current situation of people who have survived at least five years after presenting to care with AHD [36].

Despite the broad consensus on the need to measure and improve HRQoL in PLH [37], there is still limited evidence about HRQoL in long-term survivors diagnosed with AIDS or severe immunodeficiency. Longitudinal data are particularly scarce. The objectives of our study were to assess HRQoL in PLH who survived at least five years after presenting to care with AIDS or severe immunosuppression, and to identify factors that could be associated with HRQoL in the medium-to-long term after an AHD diagnosis.

## Methods

### Design

This observational and exploratory study was nested in the CoRIS cohort: an open, prospective, multicenter cohort of over 15,000 confirmed PLH. Participants were naïve to ART at study entry and were recruited in 43 Spanish hospitals from 2004 onwards. The CoRIS dataset includes baseline and follow-up sociodemographic, immunological, and clinical data. Variables collected only at inclusion were gender, age, mode of transmission, social and economic variables, country of birth, educational level, and profession. Variables collected at inclusion and yearly were AIDS events, NAEs, other comorbidities, CD4 cells/µL, HIV viral load (HIV-VL), hepatitis (B, C) coinfection, and type of ART. CoRIS data are highly standardized and are subject to periodic quality control. Participants were followed up regularly according to routine clinical practice [38]. Unfortunately, data on HRQoL were not collected during enrolment.

### Participants

**Fig. 1:**
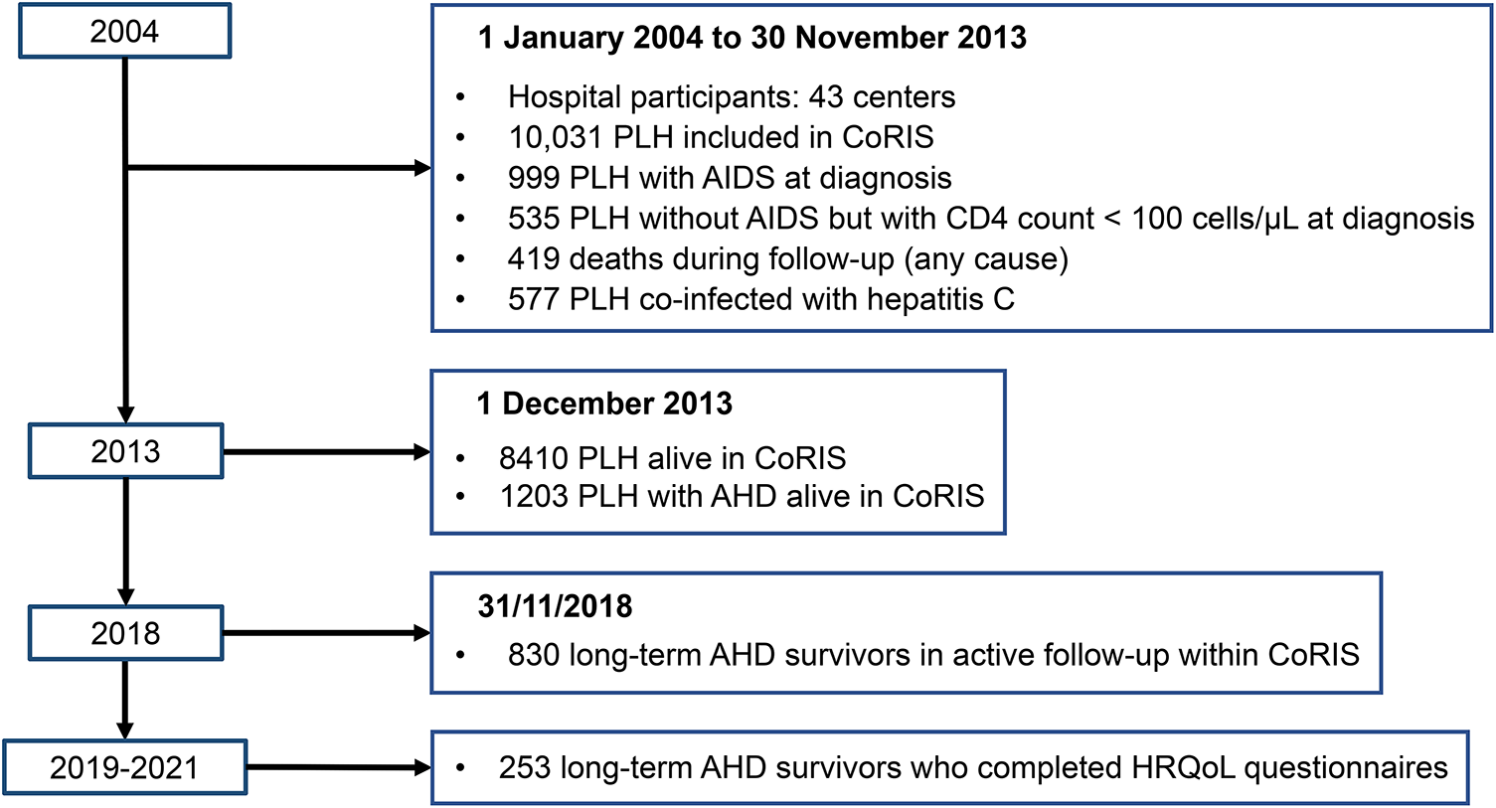
Flowchart of participant selection.* AHD* advanced HIV disease;* HRQoL* health-related quality of life;* PLH* people living with HIV

Our inclusion criteria were: (1) inclusion in the CoRIS cohort between January 2004 and November 2013, (2) survival in the cohort until November 2018, (3) age 18 years or older at inclusion in the cohort, (4) AIDS diagnosis and/or CD4 count below 100 cells/µL at inclusion, (6) completion of HRQoL questionnaires, and (7) signed informed consent. We excluded patients whose clinical data were incomplete or not updated at the time of the study (Fig. 1). All the centers with selected patients were invited to participate in the study, and they were responsible for contacting and recruiting participants.

### Study Variables and Data Collection

Exposure variables were collected at two time points: at inclusion in CoRIS (t0; baseline) and when the HRQoL questionnaires were administered (t1; long-term follow-up).

- t0
  - Sociodemographic variables: gender, age, transmission route, educational level, and place of birth.
  - Year of inclusion in the cohort.
  - Immunovirological status: CD4 cells/µL, CD8 cells/µL, CD4/CD8 ratio.
  - HIV-VL.
  - AIDS events and NAEs.
  - Type of ART: protease inhibitors (PI), non-nucleoside reverse transcriptase inhibitors (NNRTIs), integrase strand transfer inhibitors (INSTIs), and others.
- t1: time in follow-up after AHD diagnosis, age, CD4 cells/μL, CD8 cells/μL, CD4/CD8 ratio, HIV-VL, new NAEs, ART, and HRQoL scores.

We assessed HRQoL and self-perceived health using two questionnaires. These could be self-administered online or administered by an interviewer over the telephone or in a clinical setting. Data collection started in January 2020 and extended until the end of 2021 because of the disruptive effects of the COVID-19 pandemic on the Spanish health system.

The first HRQoL instrument was the WHO quality of life questionnaire for people with HIV (WHOQOL-HIV-BREF) [39], which has been validated in the Spanish population [25]. Advantages of this instrument are that it includes items specific to HIV and is more sensitive to changes in symptoms and treatment compared with generic questionnaires [40]. In addition, because it has been widely used in Spain and other countries, there is a large volume of data available to make comparisons. The scale comprises 31 items (five of which are specific to PLH) in six domains: physical health, psychological health, level of independence, social relationships, environmental health, and spirituality/religion/personal beliefs (SRPB). Each item is scored on a scale of 1 to 5. The average score for each dimension is then multiplied by 4, for a range of 4 to 20 points per dimension. Higher scores indicate better HRQoL.

We also used a generic measure of HRQoL—the EuroQol five-dimension five-level questionnaire (EQ-5D-5 L)—because the available normative data enable comparisons with the general population and other groups. The EQ-5D-5 L contains five dimensions: mobility, self-care, usual activities, pain/discomfort, and anxiety/depression. There are five possible responses or severity levels, with 1 representing the best health and 5 representing the worst health. Each participant indicates the level that best reflects his or her state for each dimension. In addition, the tool includes a 20-cm visual analogue scale (EQ-VAS) [41, 42], ranging from “The worst health you can imagine” at the bottom (score of 0) to “The best health you can imagine” at the top (score of 100). The respondents score their perceived health between the two extremes. This questionnaire has been validated in the general population of Spain [43], but not in PLH specifically.

To interpret the participants’ scores in the HRQoL measures, we used available established norms. For the WHOQOL-HIV-BREF, we referred to the study conducted in a large sample of PLH by Fuster-RuizdeApodaca and colleagues [25]. The authors followed the statistical steps for transforming direct scores into normative scores. This allows for meaningful comparisons between individuals or groups in relation to the overall population sample [44]. Fuster-RuizdeApodaca and colleagues created three category classifications: “low”, “normal”, and “high” [25]. Direct scores ranging from 16 to 18 were at the upper limit of the “normal” category in most dimensions. However, in the environmental health dimension, a direct score of 17 falls into the lower limit of the “high” category.

To interpret the EQ-5D-5 L scores, we used the norms obtained for the Spanish general population [43].

### Ethics

The study was approved by CoRIS and the ethics committee of Dr Balmis General University Hospital.

### Statistical Analysis

After data cleaning, checking statistical assumptions, and descriptive analyses, we designed the following analytical strategy. As a first step, we wanted to check for longitudinal changes in the participants’ immunological status during the study period; we anticipated a positive evolution. To test changes in the main immunological markers (CD4 cells/µL, CD8 cells/µL, and CD4/CD8 ratio), we used a general linear model (GLM) repeated measures analysis. In view of available evidence suggesting an influence of comorbidity accumulation on long-term HRQoL [45, 46], we introduced NAEs occurring during follow-up into the model as a covariate. We wanted to test the potential influence on current quality of life since evidence suggests that the accumulation of comorbidities throughout the health process may influence long-term health [45, 46].

Next, we analyzed the longitudinal influence of immunological markers at baseline (t0) and long-term follow-up (t1) on HRQoL outcomes (at t1). Logic dictates that worse health at t0 should lead to worse HRQoL at t1. However, given the minimal evidence available and the quantity and nature of the data, we used predictive models based on correlations rather than confirmatory models. Specifically, we used partial least Squares Structural Equation Modelling (PLS-SEM), which is a non-parametric technique suitable for exploratory models with small samples and which does not require distributional assumptions [47]. In PLS-SEM, relationships among the studied variables are analyzed using linear regression, wherein the loads can be interpreted as standardized beta coefficients. We tested separate models for each immunological variable at baseline, using as outcome variables the WHOQOL-HIV-BREF dimensions and the index value and EQ-VAS of the EQ-5D-5 L. The mediating role of NAEs experienced during follow-up was tested in all models, because existing evidence indicates a potential influence of comorbidities on HRQoL [45]. We wished to assess whether a causal path existed between immunological markers and HRQoL. Before starting the PLS-SEM analysis, to ascertain and control for the influence of demographic factors on participants’ current quality of life, we carried out linear regressions with each of the outcome variables (Supplementary Table 1). Next, to assess the joint contribution to the explained variance of the AIDS diagnosis on entry into the cohort, we introduced baseline immunological markers (CD4, CD8, and CD4/CD8 ratio) and NAEs into the regression models. Only educational level showed an association with some dimensions, so we controlled for this variable in the path analysis.

SmartPLS v.3.0 software [48] and the SPSS statistical package v.27 were used for data analysis.

## Results

A total of 830 CoRIS participants met our inclusion criteria. Eligible patients who accepted the invitation to participate and completed all the HRQoL questionnaires were included. The main reasons for not participating in the study (both for patients and health professionals) were directly related to the COVID-19 pandemic (e.g. work overload for HIV clinicians, suspension of visits, challenges in providing details of the study in person and obtaining informed consent). Other reasons patients gave for refusing to participate included unwillingness to use electronic devices or to provide their email address, mistrust in electronic systems, and personal reasons. A total of 176 participants (69.6%) self-administered the HRQoL questionnaires using electronic devices, while 77 participants (30.4%) provided their responses in a face-to-face interview with a healthcare professional at an HIV clinic. In most cases, the decision of whether to administer the questionnaire face-to-face was taken at the hospital level; only five patients reported difficulties using electronic devices. We performed a sensitivity analysis to compare the final HRQoL results between both administration methods, finding no significant differences between the groups. A few participants were excluded from the analysis because they made mistakes or missed some items when completing the questionnaires.

Our analysis included 253 patients with a mean age of 41.75 years (range 29 to 85 years). Most participants were male (75.5%) and white (96.8%). Regarding place of birth, 78.3% of participants were from Western Europe (including Spain), 15.8% were from South America, 3.6% from Sub-Saharan Africa, and 2.4% from other regions. The mode of transmission was through sexual relations in 88.9% of cases and through intravenous drug use in 10.3%. In terms of educational level, 15.8% of participants had completed primary studies, 66% secondary studies, and 18.2% had no data for this variable. Almost half of participants (45.9%) had an AIDS diagnosis, and all had CD4 levels below 100 cells/µL at inclusion. The most frequent AIDS events were *Pneumocystis jirovecii* pneumonia (18.8%) and tuberculosis (8.7%). The mean (± standard deviation [SD]) length of follow-up from inclusion in the cohort was 139.5 (± 33.8) months.

Table 1 describes immunovirological and clinical variables at inclusion (t0) and when HRQoL was analyzed (t1). The mean (± SD) CD4 count at inclusion was 57.6 (± 39.8) cells/µL, and the mean (± SD) CD4/CD8 ratio was 0.13 (± 0.12). Most patients started ART based on NNRTIs and boosted protease inhibitors, but by t1, over half of treatments were based on INSTIs. The most frequent NAEs detected during the study were depression and non-AIDS cancer (Table 1).

**Table 1:** Cohort characteristics at diagnosis and at ≥ 5 years of follow-up (*n* = 253)

Variables	Assessment time point	
	Diagnosis	Long-term follow-up
Age in years, mean ± SD	41.8 ± 10.2	53.4 ± 10.1
CD4 cells/µL, mean ± SD	57.6 ± 39.8	630.0 ± 327.5
CD8 cells/µL, mean ± SD	574.7 ± 392.8	889.7 ± 493.7
CD4/CD8 ratio, mean ± SD	0.13 ± 0.12	0.81 ± 0 0.44
HIV viral load		
Mean ± SD copies/mL	552,540 0.6 ± 942269.1	2054.53 ± 19151.8
< 50 copies/mL, n (%)	–	198 (78.3)
50–200 cop./mL, n (%)	–	5 (2.0)
> 200 cop./mL, n (%)	253 (100)	9 (3.6)
N/A	–	41 (16.2)
Non-AIDS events^a^, n		
Depression	6	19
Suicidality	1	1
Non-AIDS related cancer	4	15
Kidney failure	5	5
Stroke	1	5
Heart disease	1	8
Bone fracture	1	13
Osteonecrosis	0	6
Lactic acidosis	1	1
Diabetes	1	8
Liver cirrhosis	0	3
Dementia	9	2
Other	0	6
Antiretroviral treatment, n (%)		
INSTI + 2NA	9 (3.6)	124 (49.0)
Boosted PI + 2NA	107 (42.3)	38 (15.0)
NNRTI + 2NA	128 (50.6)	54 (21.3)
INSTI-NNRTI		14 (5.5)
INSTI + boosted PI		5 (2.0)
Monotherapy (boosted PI)		6 (2.4)
Other^b^	9 (3.6)	10 (4.0)
Non-ART		2 (0.8)

*ART* antiretroviral therapy;* INSTI* integrase strand transfer inhibitor;* NA* nucleoside analogue;* N/A* not available;* NNRTI* non-nucleoside reverse transcriptase inhibitor;* PI* protease inhibitors

^a^Non-AIDS events at long-term follow-up include those diagnosed at baseline

^b^Clinical trials

Table 2 shows HRQoL outcomes according to both assessment instruments. The mean scores for each WHOQOL-HIV-BREF dimension were around 15/20 points. The mean self-perceived overall health (EQ-VAS) was 76.6/100, and the mean scores in each EQ-5D-5 L dimension were under the theoretical mean of the scale and close to 1 (the best health). Table 2 shows all the descriptive statistics and the EQ-5D-5 L index value.

**Table 2:** Health-related quality of life (HRQoL) scores at ≥ 5 years of follow-up in long-term AHD survivors (*n* = 253)

Assessment instrument and dimensions	IQR		Mean ± SD
WHOQOL-HIV-BREF^a^			
General health	4.00	20.00	14.81 ± 3.43
Physical health	6.00	20.00	15.60 ± 3.31
Psychological health	6.40	20.00	15.41 ± 2.89
Level of independence	5.00	20.00	15.75 ± 3.10
Social relations	4.00	20.00	15.39 ± 3.04
Environmental health	10.00	20.00	15.73 ± 2.21
Spirituality/personal beliefs	5.00	20.00	15.45 ± 3.51
EQ-5D-5 L			
Overall health (EQ-VAS)^b^	0.00	100.00	76.57 ± 21.30
Mobility^c^	1.00	5.00	1.42 ± 0.86
Self-care^c^	1.00	4.00	1.08 ± 0.37
Usual activities^c^	1.00	5.00	1.25 ± 0.64
Pain/discomfort^c^	1.00	5.00	1.74 ± 1.06
Anxiety/depression^c^	1.00	5.00	1.68 ± 0.91
Sum score dimensions EQ-5D	5.00	24.00	7.17 ± 3.00
EQ-5D-5 L index value	−1	1	0.85 ± 0.21

*AHD* advanced HIV disease;* EQ-5D-5* EuroQol 5-dimension 5-level quality of life questionnaire;* IQR* interquartile range;* SD* standard deviation

^a^The score ranges from 4 to 20 for each dimension, with higher scores indicating better HRQoL

^b^The score ranges from 0 (worst overall health) to 100 (best overall health)

^c^The score ranges from 1 (representing the best health) to 5 (representing the worst health)

The GLM repeated measures analysis showed a significant increase from t0 to t1 in mean (± SD) CD4 count (57 ± 39.07 cells/µL vs. 629 ± 331 cells/µL; F[1,209] = 585.83; *p* < 0.001; ɳ2 = 0.719), in CD8 count (542 ± 291 cells/µL vs. 935 ±:416.07 cells/µL; F[1,101] = 62.62; *p* < 0.001; ɳ2 = 0.383), and in CD4/CD8 ratio (0.12 ± 0.13 vs. 0.79 ± 0.42; F[1,132] = 310.95; *p* < 0.001; ɳ2 = 0.702). There was a marginally positive CD4/CD8 ratio associated with the covariate NAEs (F[1,132] = 3.65; *p* = 0.058). NAEs did not significantly covary with either CD4 or CD8 longitudinal changes.

Regarding the influence of immunological markers at baseline (t0) on HRQoL outcomes (t1), we found the following results. The separate regression analyses showed that most models were non-significant. The model with the dependent variable physical health showed borderline significance (F[8, 130] = 1.80; *p* = 0.078). In this model (R^2^ = 0.09), significant variables were higher level of education (Beta = 0.23, *t* = 0.05, *p* = 0.010) and fewer NAEs during follow-up (Beta = -0.20, *t* =–2.36, *p* = 0.019). No other demographic or immunological variable was significant. The ANOVA of the regression with the dependent variable psychological health was also borderline significant (*F* [8, 130] = 1.93 *p* = 0.069). In this model (R^*2*^ = 0.09), the only significant variable was level of education (Beta = 0.19, *t* = 2.23, *p* = 0.027), while borderline significant variables were CD8 count at t0 (Beta = 0.18, *t* = 1.96, *p* = 0.053) and number of NAEs during follow-up (Beta = − 0.15, *t* =–1.78, *p* = 0.077). The regression models with the remaining WHOQOL dimensions were non-significant. However, CD4 count was a borderline significant predictor of environmental health (Beta =–0.22, *t* =–1.95, *p* = 0.053).

Regarding the EQ-5D-5 L, although the regression analysis of index value was non-significant (F[8, 130] = 1.60 *p* = 0.14), number of NAEs did show statistical significance (Beta =–0.20, t =–2.32, *p* = 0.022). The model explained a low variance, as in the previous cases (R^2^ = 0.08). Neither the model nor the predictors were significant in the EQ-VAS analysis.

**Fig. 2:**
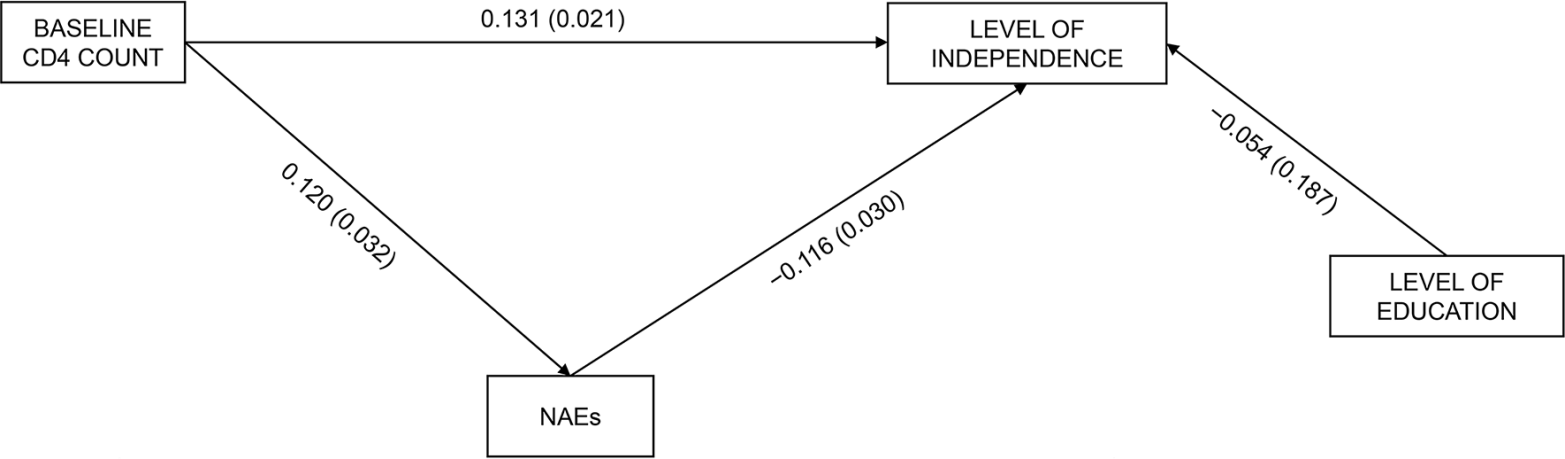
Influence of baseline CD4 count on level of independence dimension in the WHOQOL-HIV-BREF health-related quality of life scale at long-term follow-up. standardized β (p value).* NAES* non-AIDS events;* WHOQOL-HIV-BREF* World Health Organization quality of life questionnaire for people living with HIV

**Fig. 3:**
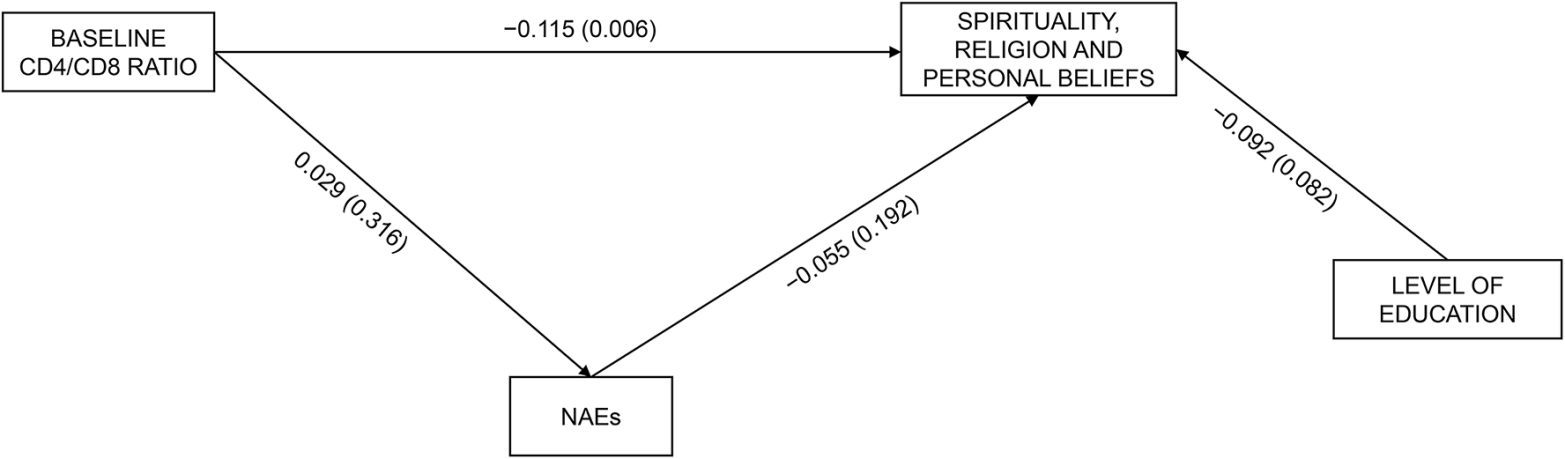
Influence of baseline CD4/CD8 ratio on spirituality, religion and personal beliefs dimension of the WHOQOL-HIV-BREF at long-term follow-up. Standardized β (p-value).* NAES* non-AIDS events;* WHOQOL-HIV-BREF* World Health Organization quality of life questionnaire for people living with HIV

Next, we tested the PLS-SEM separated models analyzing for each immunological variable at baseline, considering the WHOQOL-HIV-BREF dimensions and the EQ-5D-5 L index value and EQ-VAS as outcome variables. Supplementary Tables 2 and 3 present the results of the PLS models analyzed using the various WHOQOL-HIV-BREF dimensions as the dependent variables. Figures 2 and 3 present the models that showed significant relationships. We found a positive association between CD4 count at t0 and score on the independence dimension at t1 (R^2^ = 0.030). However, a higher baseline CD4 count was associated with a higher number of NAEs, which in turn predicted a lower level of independence in study participants. This means NAEs partially mediated between baseline CD4 and current level of independence; however, the specific indirect effects of baseline CD4 on the level of independence were non-significant (indirect effect = − 0.014, t − 1.35, *p* = 0.175). There were no significant associations between CD8 at t0 and any HRQoL dimensions or number of NAEs. Finally, higher CD4/CD8 ratios negatively predicted the score on the SRPB dimension (R^2^ = 0.023). NAEs played no role in this relationship.

**Fig. 4:**
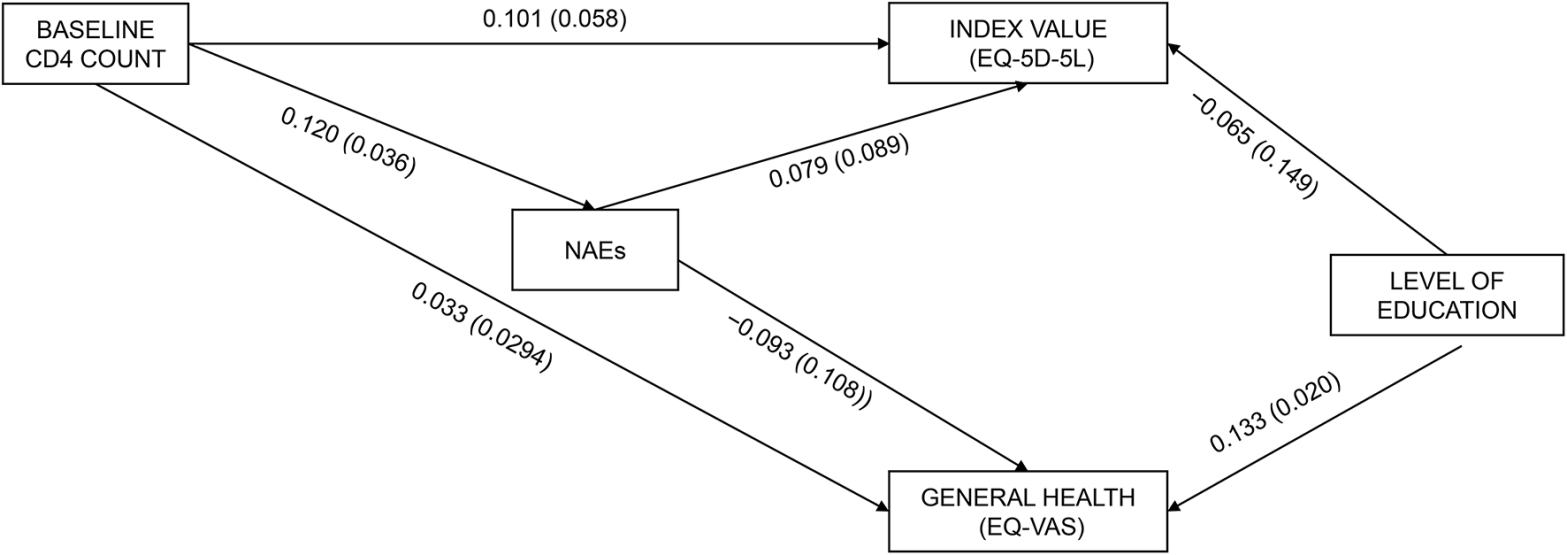
Influence of baseline CD4 count on EQ-5D-5L Index Value and Global Health (EQ-VAS) at long-term follow-up. Standardized β (p-value).* EQ-5D-5L* EuroQol 5-dimensión 5-level quality of life questionnaire;* EQ-VAS* EuroQol visual analogue scale;* NAEs* non-AIDS events

**Fig. 5:**
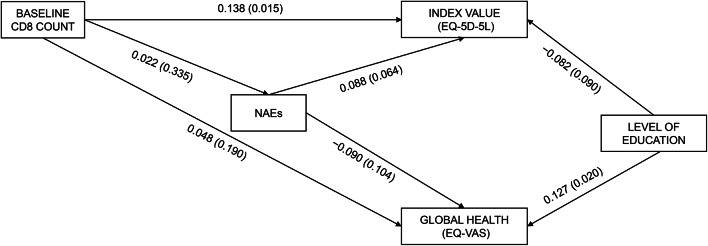
Influence of baseline CD8 count on the EQ-5D-5L Index Value and Global Health (EQ-VAS) at long-term follow-up. Standardized β (p-value).* EQ-5D-5L* EuroQol 5-dimensión 5-level quality of life questionnaire;* EQ-VAS* EuroQol visual analogue scale;* NAEs* non-AIDS events

Figures 4 and 5 show the results of the models that used EQ-5D-5 L scores as dependent variables. In addition, supplementary Tables 4 and 5 present the results of the PLS models analyzed using the various EQ-5D-5 L dimensions as the dependent variables. CD4 count at t0 positively predicted the EQ-5D-5 L index value (R^2^ = 0.022) with a marginally significant relationship but was not associated with general health perception (R^2^ = 0.026). There was also a significant positive association between CD4 count and number of NAEs; however, NAEs were not significantly related to either HRQoL variable. CD8 at t0 was a positive and significant predictor of the EQ-5D-5 L index value (R^2^ = 0.030). The only other variable in the model to show a significant relationship with HRQoL was educational level, which was directly associated with general health perception. We found no significant associations between CD4/CD8 ratio and the dependent variables.

## Discussion

The descriptive results of our study suggest that in the new era of ART, among PLH living in a European country who have AHD at HIV diagnosis and who survive at least five years, HRQoL is moderate or “normal” according to the available references norms established in Spanish PLH and the general Spanish population [25, 43]. Thus, there is some room for improvement. We selected five years as a minimum survival time based on the methods of similar studies in cancer survivors [36, 49, 50]. Outcomes were similar for both HRQoL tests used in this study. Of note, the scores for different WHOQOL-HIV-BREF dimensions were all similar, although they were slightly higher than in other studies performed in the Spanish HIV population [25] and elsewhere [51, 52, 53]. In Spain, Fuster Ruiz-de-Apodaca and colleagues studied 1462 PLH with different clinical and socioeconomical conditions [25]. The mean (± SD) HRQoL scores of this general PLH population in Spain and the AHD survivors in our study are comparable: general health, 14.9 ± 3.6 versus 14.81 ± 3.43; physical health, 15.5 ± 3.2 versus 15.60 ± 3.31; psychological health, 14.9 ± 3.0 versus 15.41 ± 2.89; level of independence, 15.5 ± 3.2 versus 15.75 ± 3.10; environmental health, 15.3 ± 2.5 versus 15.73 ± 2.21; and SRPB, 14.5 ± 3.5 versus 15.45 ± 3.51.

Our analysis based on EQ-5D-5 L scores in long-term ADH survivors showed worse outcomes than those reported in the general Spanish population, although great caution is warranted when interpreting this difference in view of the small sample size of our study. On average, the Spanish population reference value for the EQ-VAS (general health) in the age range of our sample is 81.4 points, compared with 76.57 (± 21.30) points in our cohort. Regarding the EQ-5D-5 L index value, the overall normative reference reported in the Spanish population in the age range of our participants is 0.96 (± 0.11) points, compared with 0.85 (± 0.21) points in our cohort [54]. Similarly, PLH receiving ART in the UK have lower EQ-5D-5 L scores than the general UK [55]. Although comparisons are complicated by the difference in sample size and epidemiological characteristics, one potential explanation is that the characteristic of long-term survival may translate to greater resilience, which could impact some dimensions of HRQoL. Other studies have found post-traumatic growth in PLH with a long history of coping, resilience, and activism [27, 56].

In the longitudinal analyses, as expected, we found an improvement in the immunological markers during follow-up, reflecting an important immune recovery. Baseline immunological markers influenced some WHOQOL-HIV-BREF dimensions, albeit modestly. For example, there was a positive association between CD4 count at t0 and level of independence at t1. In addition, the results of the models showed that both CD4 count and CD8 count at t0 had a positive impact on the EQ-5D-5 L index value; however, higher CD4/CD8 ratios at t0 were associated with lower scores on the WHOQOL-HIV-BREF SRPB dimension at t1. Although this result seems contradictory, one tentative hypothesis is that PLH with AHD at diagnosis and with better immunological status have faced fewer physical and psychological health challenges over the years, so have required less post-traumatic growth and resilience than PLH with poorer baseline health. We observed that NAEs may play a mediating role in the relationship between CD4 cell count and certain HRQoL variables. This is important because PLH with AHD are more likely to develop NAEs than those diagnosed earlier [57].

Previous studies have shown that PLH with an AIDS diagnosis or very low CD4 counts present lower scores on different HRQoL dimensions, especially physical health [13, 25] and psychological health [15, 27, 28]. Only a few longitudinal studies have analyzed changes in HRQoL after an AHD diagnosis. Anis and colleagues, from the Clinical Optimal Trial (2001–2007) [45], reported that higher CD4 counts were associated with a significant increase in HRQoL scores, especially in physical and psychological health. One publication from the Multicenter AIDS Cohort Study (2001–2004) reported that an AIDS diagnosis by itself was no longer significantly related to scores on the physical health dimension after controlling for all collected variables, such as HIV symptoms and treatment of opportunistic infections [58]. To the best of our knowledge, ours is the first study to analyze HRQoL in long-term AHD survivors (≥ 5 years) in the modern ART era.

Our longitudinal analyses suggest a role for NAEs in both immunological recovery and HRQoL. The evolution of immunological markers over follow-up (linear repeated measures analyses) showed a marginally positive covariation between number of NAEs during follow-up period and increase in CD4/CD8 ratio. Furthermore, the occurrence of NAEs had an indirect negative effect on level of independence as measured by the WHOQOL-HIV-BREF. Previous studies have associated a low nadir CD4 count, a low CD4/CD8 ratio, and CD4/CD8 recovery with a higher incidence of NAEs and mortality [57, 59, 60]. NAEs in late-presenters have been related to increased inflammation and immune activation associated with immune recovery [61]. Other studies, mainly cross-sectional, have linked immune status to HRQoL [12, 25].

In our study, the only demographic variable that showed a positive association with some HRQoL dimensions was educational level. Education can influence access to socioeconomic resources and medical services, and this access, in turn, may influence HRQoL dimensions such as level of independence and index value of global health. Previous research has pointed to education as a determinant of HRQoL [62].

Our study has some limitations. First and foremost, we were unable to assess change in HRQoL from baseline to follow-up, because participants provided no quality of life data at the time of AHD diagnosis. Consensus statements on the importance of evaluating quality of life are relatively recent [37, 63], as they stem from advances in the care model and the evolving nature of the disease [64]. Until the late 1990s, patient-reported measures in PLH focused more on risk behaviors, adherence, or symptoms; quality of life was measured mainly in the context of clinical trials [65, 66]. However, there is evidence to suggest average quality of life was bad or very bad in this population [12, 14, 24]. Although our study included no assessment of change in HRQoL owing to lack of baseline data, our follow-up data can inform future research on HRQoL evolution and its impact on the long-term health of PLH.

Second, we evaluated a single arm of long-term AHD survivors; our study included no comparative arm of PLH without AHD. A wide range of clinical and socioeconomic conditions can affect HRQoL in PLH, and most are covered in the scientific literature [12, 24, 25, 45]. Our objective was to determine which baseline conditions may be related to worse HRQoL in a specific and under-researched population: people who survive at least five years after presenting to care with AHD. We decided to compare our results with those of a gender- and age-matched sample of the Spanish general population.

Third, this study was carried out during the successive waves of COVID-19 in Spain, which we worried could have a negative impact on HRQoL. Nevertheless, even during the COVID-19 pandemic, HRQoL scores in our cohort were comparable with the normative data of the general Spanish population. On the other hand, the pandemic did reduce participation, both among PLH and hospitals.

Fourth, because we were unable to collect HRQoL data from individuals who died or were lost to follow-up during the study, our results may be affected by survivorship bias and have limited generalizability. Although our study used adequate techniques (PLS-SEM) to analyze predictive models, the small sample size and the nature of the variables led to some limitations, such as the low proportion of variance explained. However, it is more important to build theory than data [67], and it is reasonable to assume that a retrospective immunological marker has only a relative contribution to a complex multidimensional construct such as HRQoL.

Of the broad range of HRQoL determinants in PLH [62], we were able to analyze the role of immunological markers and non-AIDS-related comorbidities in our study. We also tested the influence of some sociodemographic factors, such as sex, educational level, and socioeconomic status. However, we were unable to analyze important behavioral factors such as substance use, lifestyle, or treatment adherence. Nor could we measure the contribution of highly relevant psychological factors such as mental health, social support, and stigma [68]. Despite the limitations of our analysis, it is important to emphasize the importance of reporting them [69, 70]. Reporting variables that are non-significant or that have very low explained variance guarantees scientific progress, as these valuable data help researchers avoid biases and optimize subsequent analyses and resources. They enrich knowledge, promote innovation and critical thinking, and help to redefine paradigms, question assumptions, and stimulate scientific collaboration. Research and clinical practice must move towards a patient-centered model based on variables that influence HRQoL in PLH as they live and cope with the challenges associated with their disease [37].

Our study also has strengths. First, it was nested in the CoRIS cohort, which has a large study population, is highly comprehensive, uses robust methods to collect sociodemographic and clinical data, and, most notably, has a very long follow-up. Another strength was our objective of studying HRQoL in long-term AHD survivors, which could explain the strong HRQoL outcomes. Different cohort studies have shown that the highest risk in people with AHD is present during the first two years after diagnosis [35, 71, 72, 73].

## Conclusions

Overall, our study shows that in the modern ART era, people living in Spain who have AIDS or severe immunodeficiency at HIV diagnosis and who remain in clinical care at least five years later achieve moderate levels of HRQoL in all domains, but with room for improvement. This vulnerable population continues to carry a high risk of mortality due to AIDS events and NAEs, at least during the first two years after diagnosis [45, 74]. The antiretroviral treatment guidelines from WHO [75] and from the British HIV Association [76] define AHD as a special state in the spectrum of HIV disease. However, both guidelines focus on the antiretroviral treatment and prevention of opportunistic diseases. Owing to the high risk of NAEs and mortality in PLH with AHD, this population requires special resources to improve life expectancy and long-term HRQoL [19, 20, 21, 22, 23]. Relevant measures include: (1) screening for and interventions to reduce traditional risk factors of NAEs, especially for non-cancer AIDS; (2) creating sustainable care models to implement these interventions during follow-up; (3) ensuring treatment adherence; (4) screening for mental health issues and providing the necessary psychological support; and (5) increasing the frequency of clinical visits and personalized clinical care based on socioeconomic inequities, with special attention on vulnerable populations. In our opinion, HIV guidelines should consider AHD patients as a special population whose needs are different from PLH who present for care soon after an HIV infection.

## Supplementary Information

Below is the link to the electronic supplementary material.

Supplementary Material

## Data Availability

Dataset is available upon request.
