# Supplementary material for: Long-Term Health-Related Quality of Life in People Living with HIV Who Present to Care with AIDS or Severe Immunodeficiency: The CoRIS AIDS Survivors Study
Source: AIDS Behav. 2025 May 15;29(9):2729–41. doi: 10.1007/s10461-025-04730-x (PMC12432043; doi:10.1007/s10461-025-04730-x)
Supplement: Supplementary file 1 — Supplementary Material [file 10461_2025_4730_MOESM1_ESM.docx]

Supplementary Table 1. Coefficients of linear regression assessing the influence of demographics (t0) on the different domains of HRQoL in the BRIEF_WHOQoL questionnaire

|  | General Health | | Physical Health | | Psychological Health | | Level of Independence | | Environmnental  health | | Social relationships | | SRPB | |
| --- | --- | --- | --- | --- | --- | --- | --- | --- | --- | --- | --- | --- | --- | --- |
|  | Beta | t (p) | Beta | t (p) | Beta | t (p) | Beta | t (p) | Beta | t (p) | Beta | t (p) | Beta | t (p) |
| Age | -0.02 | -0.08 (.94) | 0.09 | 0.39 (.70) | -0.20 | -0.90 (.38) | -0.06 | -0.27 (.79) | 0.02 | 0.08 (.94) | -0.08 | -0.35 (.73) | 0.23 | 1.05 (.31) |
| Gender | 0.05 | 0.20 (.85) | 0.19 | 0.73 (.48) | 0.17 | 0.69 (.50) | -0.12 | -0.50 (.62) | -0.11 | -0.42 (.68) | 0.17 | 0.70 (.49) | 0.25 | 1.02 (.32) |
| Mode transmission | -0.24 | -1.31 (.21) | -0.19 | -0.82 (.42) | -0.13 | -0.58 (.57) | -0.23 | -1.10 (.28) | -0.06 | -0.24 (.82) | -0.37 | -1.70 (.11) | -0.21 | -0.96 (.35) |
| Level education | -0.71 | -2.65  (.02) | -0.25 | -0.95  (.35) | -0.27 | -1.05 (.31) | -0.57 | -2.38 (.03) | -0.28 | -1.02 (.32) | -0.24 | -0.98 (.34) | -0.22 | -0.88 (.39) |
| Employement | 0.04 | 0.23 (.82) | 0.03 | 0.12 (.90) | 0.24 | 1.04 (.31) | -0.02 | -0.08 (.94) | 0.22 | 0.90 (.38) | 0.17 | 0.77 (.45) | -0.03 | -0.12 (.90) |
| R^2^ | .47 | | .15 | | .20 | | .31 | | .09 | | .24 | | .22 | |
| F(df); p | 2.40 (5.17); .075 | | 0.61 (5.17); .694 | | 0.85 (5.17); .531 | | 1.51 (5.17); .236 | | 0.33 (5.17); .884 | | 1.05 (5.17); .422 | | 0.99 (5.17); .453 | |

HRQoL = health-related quality of life. SRPB = Spirituality, Religion and Personal Beliefs.

Supplementary Table 2. Influence of CD4 count at baseline (t0) on the level of independence domain: results of bootstrapping and model fit.

|  | T statistics | p-values | Confidence intervals bias corrected (5%-95%) | Estimated model |
| --- | --- | --- | --- | --- |
| CD4 t0 -> NAES | 1.822 | 0.034 | 0.010 0.220 |  |
| CD4 t0 -> Level Independence | 2.042 | 0.021 | 0.022 0.231 |  |
| NAES -> Level Independence | 1.879 | 0.030 | -0.210 -0.007 |  |
| Education -> Level Independence | 0.899 | 0.184 | -0.155 0.044 |  |
| **Model Fit Statistics** |  |  |  |  |
| SRMR |  |  |  | 0.012 |
| d_ULS |  |  |  | 0.001 |
| D_G |  |  |  | 0.000 |
| Chi-Square |  |  |  | 0.353 |
| NFI |  |  |  | 0.970 |

NAES: non-AIDS events; SRMR: Standardized Root Mean Squared Residual; d-ULS: diagonally and Unweighted Least Squares; D_G: Geodesic_Distance; NFI: Normed Fit Index.

Supplementary Table 3. Influence of rate CD4/CD8 t0 in the Spirituality, Religion and Personal Beliefs (SRPB): results of bootstrapping and model fit.

|  | T statistics | p-values | Confidence intervals bias corrected (5%-95%) | Estimated model |
| --- | --- | --- | --- | --- |
| Rate CD4_CD8 t0 -> SRPB | 2.493 | 0.006 | -0.191 -0.040 |  |
| Rate CD4_CD8 t0 -> NAES | 0.478 | 0.316 | -0.067 0.129 |  |
| Education -> SRPB | 1.391 | 0.082 | -0.201 0.017 |  |
| NAES -> SRPB | 0.871 | 0.192 | -0.160 0.050 |  |
| **Model Fit Statistics** |  |  |  |  |
| SRMR |  |  |  | 0.014 |
| d_ULS |  |  |  | 0.002 |
| D_G |  |  |  | 0.000 |
| Chi-Square |  |  |  | 0.479 |
| NFI |  |  |  | 0.952 |

NAES: non-AIDS events; SRPB = Spirituality, Religion and Personal Beliefs; SRMR: Standardized Root Mean Squared Residual; d-ULS: diagonally and Unweighted Least Squares; D_G: Geodesic_Distance; NFI: Normed Fit Index.

Supplementary Table 4. Influence of CD4 count at baseline (t0) in the EQ-5D-5L Index Value and the self-perception of health (EQ-VAS): results of bootstrapping and model fit.

|  | T statistics | p-values | Confidence intervals bias corrected (5%-95%) | Estimated model |
| --- | --- | --- | --- | --- |
| CD4 t0 -> EQ Index Value | 1.570 | 0.058 | -0.007 0.207 |  |
| CD4 t0 -> EQVAS (Perception Health) | 0.543 | 0.294 | -0.059 0.135 |  |
| CD4 t0 -> NAES | 1.797 | 0.036 | 0.015 0.229 |  |
| Education -> EQ Index Value | 1.039 | 0.149 | -0.165 0.035 |  |
| Education -> EQVAS (Perception Health) | 2.049 | 0.020 | 0.029 0.235 |  |
| NAES -> EQ Index Value | 1.346 | 0.089 | -0.016 0.176 |  |
| NAES -> EQVAS (Perception Health) | 1.239 | 0.108 | -0.210 0.037 |  |
| **Model Fit Statistics** |  |  |  |  |
| SRMR |  |  |  | 0.040 |
| d_ULS |  |  |  | 0.024 |
| D_G |  |  |  | 0.005 |
| Chi-Square |  |  |  | 6.357 |
| NFI |  |  |  | 0.714 |

EQVAS: EuroQol visual analogue scale; NAES: non-AIDS events; SRMR: Standardized Root Mean Squared Residual; d-ULS: diagonally and Unweighted Least Squares; D_G: Geodesic_Distance; NFI: Normed Fit Index.

Supplementary Table 5. Influence of CD8 count at baseline (t0) in the EQ-5D-5L Index Value and the self-perception of health (EQ-VAS): results of bootstrapping and model fit.

|  | T statistics | p-values | Confidence intervals bias corrected (5%-95%) | Estimated model |
| --- | --- | --- | --- | --- |
| CD8 t0 -> EQ Index Value | 2.172 | 0.015 | 0.029 0.238 |  |
| CD8 t0 -> EQVAS (Perception Health) | 0.879 | 0.190 | -0.047 0.128 |  |
| CD8 t0 -> NAES | 0.425 | 0.335 | -0.059 0.110 |  |
| Education -> EQ Index Value | 1.342 | 0.090 | -0.180 0.020 |  |
| Education -> EQVAS (Perception Health) | 2.048 | 0.020 | 0.023 0.226 |  |
| NAES -> EQ Index Value | 1.527 | 0.064 | -0.015 0.176 |  |
| NAES -> EQVAS (Perception Health) | 1.257 | 0.104 | -0.219 0.014 |  |
| **Model Fit Statistics** |  |  |  |  |
| SRMR |  |  |  | 0.040 |
| d_ULS |  |  |  | 0.025 |
| D_G |  |  |  | 0.005 |
| Chi-Square |  |  |  | 6.676 |
| NFI |  |  |  | 0.749 |

EQ: EuroQoL; EQVAS: EuroQol visual analogue scale; NAES: non-AIDS events; SRMR: Standardized Root Mean Squared Residual; d-ULS: diagonally and Unweighted Least Squares; NFI: Normed Fit Index; D_G: Geodesic_Distance;
